# A Fatal Case of Strangulated Pericecal Hernia

**DOI:** 10.7759/cureus.16097

**Published:** 2021-07-01

**Authors:** Ahmed Bensaad, Jihane Habi, Karim El Aidaoui, Abdelaziz Fadil, Khalid Sair

**Affiliations:** 1 General Surgery, Cheikh Khalifa International University Hospital, Mohammed VI University of Health Sciences, Casablanca, MAR; 2 Radiology, Cheikh Khalifa International University Hospital, Mohammed VI University of Health Sciences, Casablanca, MAR; 3 Intensive Care Unit, Cheikh Khalifa International University Hospital, Mohammed VI University of Health Sciences, Casablanca, MAR; 4 Emergency Surgery, Cheikh Khalifa International University Hospital, Mohammed VI University of Health Sciences, Casablanca, MAR

**Keywords:** pericecal hernia, internal hernia, small-bowel obstruction, exploratory laparotomy, septic shock

## Abstract

Pericecal hernia, a subtype of internal hernia, is a rare and unusual cause of small-bowel obstruction (SBO). We report the case of a pericecal hernia in an 80-year-old woman who presented to the emergency department with symptoms of SBO. She experienced colicky diffuse abdominal pain associated with vomiting and obstipation for over five days. Abdominal examination showed rebound tenderness in the right iliac fossa without any mass palpation.

Computed tomography noted a “sac-like” structure in the right iliac fossa with dilated and ischemic small-bowel loops within. Exploratory laparotomy showed strangulated pericecal hernia and non-viable small bowel loops in the inferior ileocecal recess. Extensive resection and defect repair followed by small-bowel end-to-end primary anastomosis was made. After two days of ICU admission, patient died from septic shock.

This case report highlights the need for prompt diagnosis, followed by surgical intervention to lower mortality of SBO of internal hernia origin, especially in cases where no previous surgery is noted.

## Introduction

Internal hernias are protrusions of viscera, generally small bowel loops, through mesenteric or peritoneal defects. Cases of small bowel obstruction (SBO) due to internal hernia, and specifically pericecal hernia, are rare, with mortality of up to 50% reported in complicated cases [1]. It represents a challenging and difficult diagnosis to clinicians due to the non-specific clinical findings [2]. Strangulation of the herniated content may lead to necrosis, which makes complicated pericecal hernia a life-threatening and serious condition [3]. This case report highlights the lethal prognosis of a delayed diagnosis and stresses the need to consider pericecal hernia in the differential diagnosis of SBO in patient with no previous history of surgery or trauma.

## Case presentation

**Figure 1:**
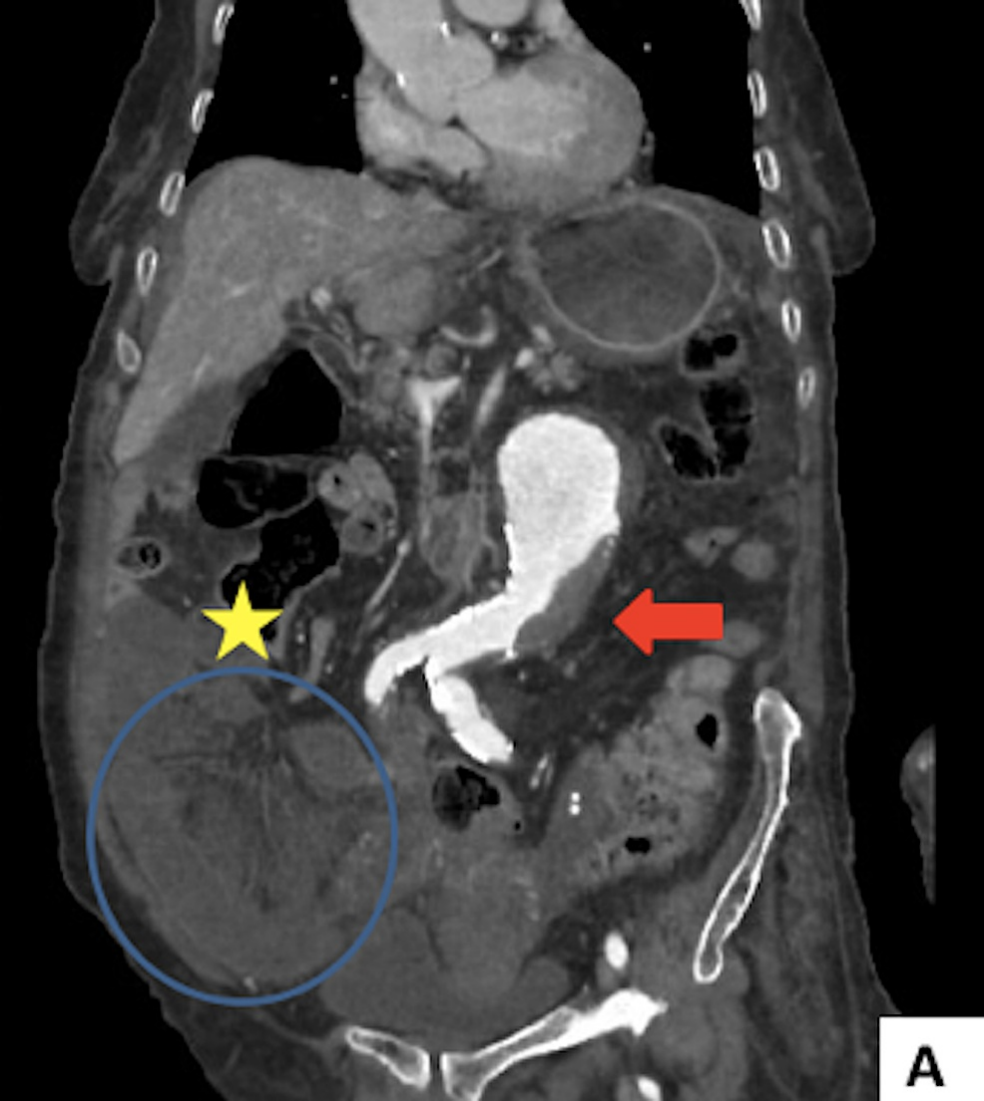
Coronal (A) CT scans demonstrating strangulated small-bowel loops in a “sac-like” formation (blue circle), shifting the ascending colon (yellow star) upward and thombosed abdominal aorta aneurysm (red arrow).

**Figure 2:**
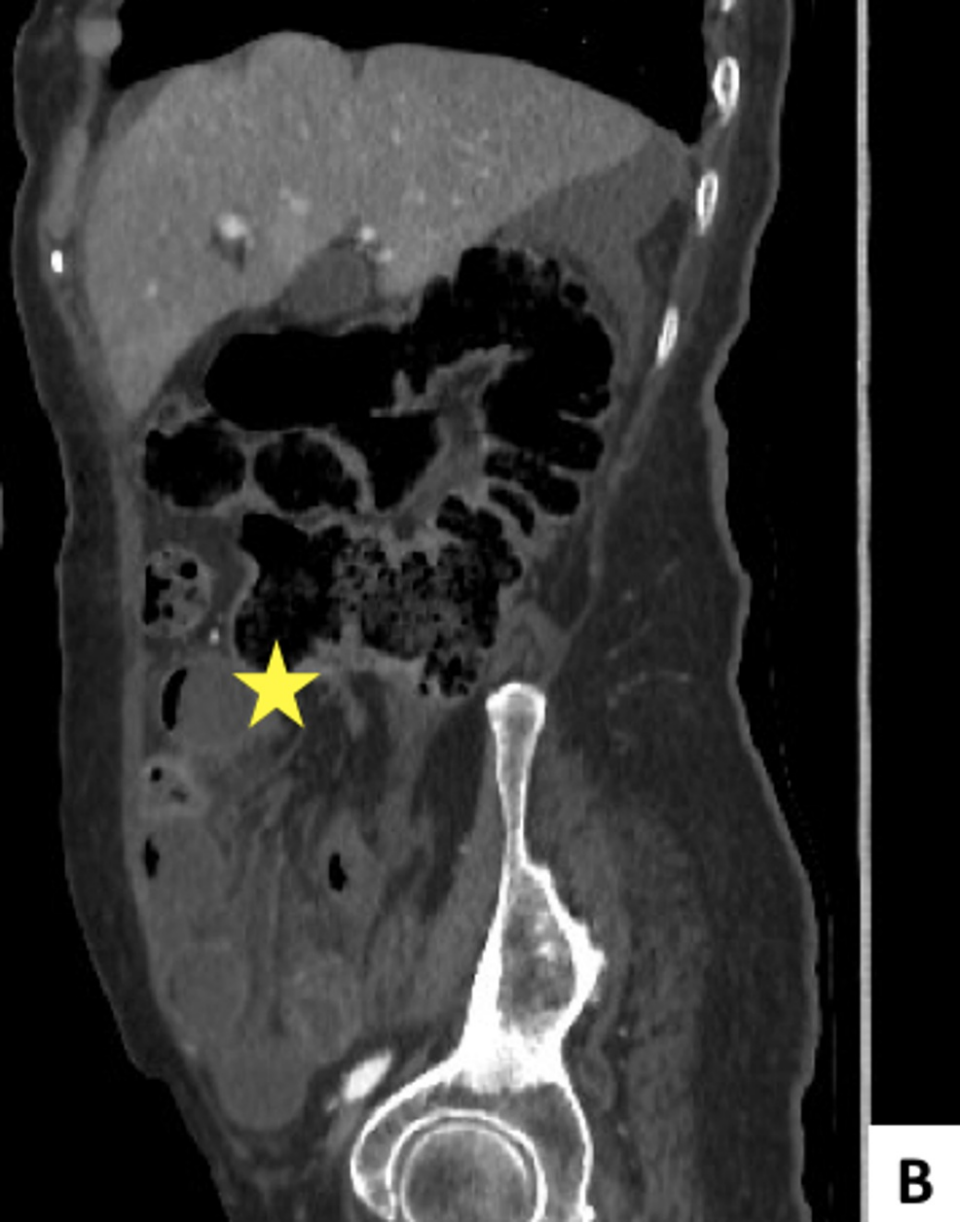
Sagittal (B) CT scans demonstrating strangulated small-bowel loops in a “sac-like” formation, shifting upward the ascending colon (yellow star).

We report the case of an 80-year-old woman, with a known history of type II diabetes, who presented to the Emergency Department for colicky diffuse abdominal pain, associated with vomiting and obstipation for over five days. No previous surgery was noted in her past medical record. She reported no previous tobacco or alcohol use. On examination, patient was dehydrated, Glasgow coma scale score of 15/15, temperature of 37,8°C, respiratory rate of 26 breaths per ​minute, blood pressure of 108/78 and a pulse rate of 110 bpm. On abdominal examination, no previous surgical scar was noted on inspection, and palpation revealed right lower quadrant and hypogastric rebound tenderness without any palpable mass. Laboratory exams showed elevated white blood cell amount of 18000/mm3 and elevated C-reactive protein of 57mg/l. Abdominal CT scan showed a “sac-like” structure in right iliac fossa measuring 148x64mm, with herniated, distended and ischemic small-bowel loops within. Ascending colon was seemingly pushed upward by the herniated content (Figures 1, 2).

**Figure 3:**
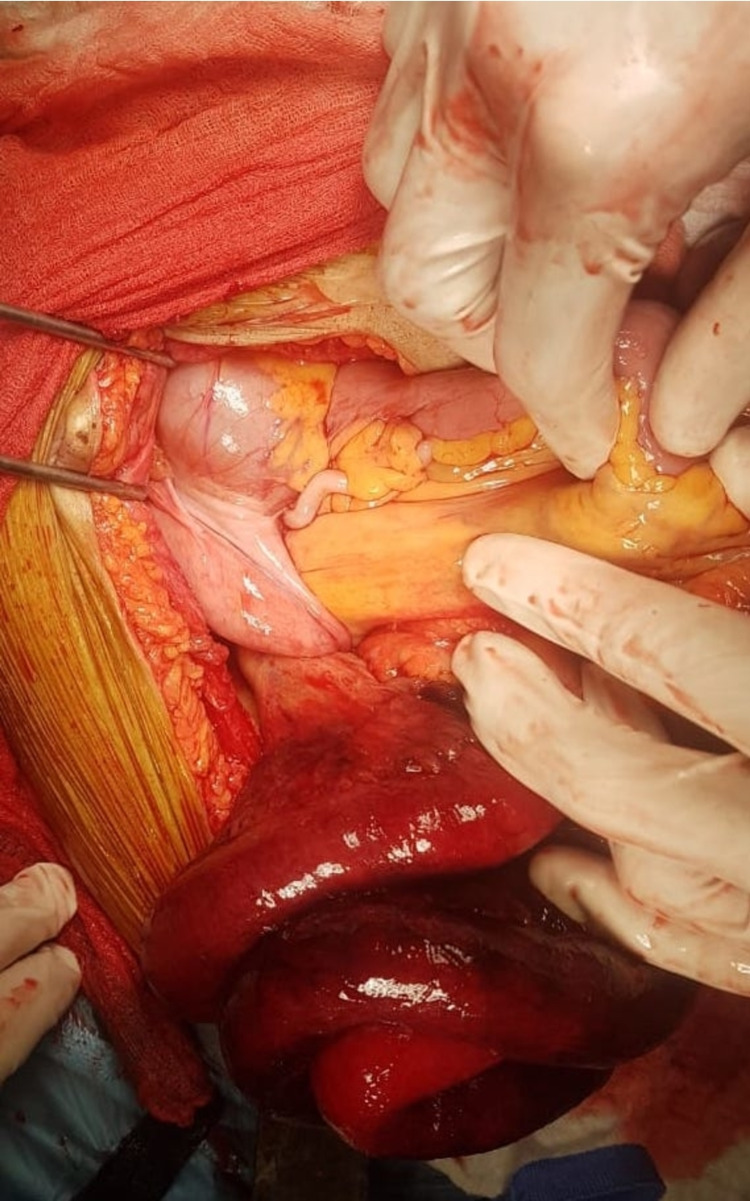
Operative view of herniated small-bowel loops and mesentery in the inferior ileocecal recess during exploratory laparotomy.

Exploratory laparotomy was indicated and showed evidence of internal hernia with strangulated small-bowel loops in inferior ileocecal recess (Figure 3).

Small-bowel was necrotic and non-viable after resuscitation attempt. Segmental resection of 110cm of distal ileum 20cm proximal to the caecum followed by small-bowel end-to-end primary anastomosis was done and the defect was closed. On histopathology, report showed changes compatible with ischemic necrosis. In the postoperative course, the patient presented a shock state. She was admitted to ICU and was given norepinephrine in association to intravenous fluid resuscitation and antibiotic therapy. Unfortunately, the patient died from septic shock with multiorgan failure two days after ICU admission.

## Discussion

Internal hernias (IH) are defined as protrusions of viscera through mesenteric or peritoneal defects. IH are mainly asymptomatic with an estimated incidence of up to 2%. Almost 5.8% of SBO are thought to be due to internal hernia; pericecal hernia may cause SBO in rare cases and form about 13% of all internal hernia [4]. Pathogenesis of different pericecal fossae in patients with no previous surgery may be explained by diverse factors, among which incomplete intestinal rotation and retroperitoneal fixation together with age-related tissue weakening. Four pericecal recesses are classically described: retrocecal recess, paracolic sulci, superior ileocecal recess, and inferior ileocecal recess. Protrusion of small bowel loops in the case described herein occurred through the inferior ileocecal recess [5]. Internal hernia, including pericecal hernia, poses a real diagnostic challenge to physicians. Most frequently, patients present with symptoms of SBO. Severity of symptoms depends on timing of presentation. Localized rebound tenderness may be noted on physical examination in early stages. Computed tomography (CT) is the key imaging technique to establish the diagnosis and to indicate the need for surgical exploration. Classical CT aspect is an “agglomeration” in sac-like form of small-bowel loops, lateral to the cecum, shifting the ascending colon upward and medially [6]. Surgery is mandatory in SBO cases that are thought to be of internal hernia origin. Laparotomy is the preferred approach, especially in late-diagnosed cases. Laparoscopic exploration and treatment may be feasible in some cases, especially after decompression of small-bowel loops to avoid unnecessary injuries [7,8]. 

## Conclusions

Strangulated pericecal hernia is a rare and challenging condition, with high mortality rate. Our case report highlights the necessity of a prompt diagnosis. Immediate intervention should be carried out to avoid necrosis of the herniated content, once the diagnosis is suspected on clinical and radiological evaluation.
